# Outcomes in Treatment-Naïve Patients With Metastatic Extremity Osteosarcoma Treated With OGS-12, a Novel Non–High-Dose Methotrexate–Based, Dose-Dense Combination Chemotherapy, in a Tertiary Care Cancer Center

**DOI:** 10.1200/JGO.17.00137

**Published:** 2018-02-08

**Authors:** Jyoti Bajpai, Arun Chandrasekharan, Vijai Simha, Vikas Talreja, Ashay Karpe, Nikihil Pandey, Ashish Singh, Bharat Rekhi, Tushar Vora, Jaya Ghosh, Shripad Banavali, Sudeep Gupta

**Affiliations:** All authors: Tata Memorial Hospital, Mumbai, India.

## Abstract

**Purpose:**

Metastatic osteosarcoma is largely treated with high-dose methotrexate (HDMTX)–based therapy, especially in the pediatric population. This mandates complex pharmacokinetic monitoring in a costly inpatient setting to mitigate unpredictable serious toxicities. Hence, a non-HDMTX–based regimen is worth exploring, especially in India and low- and middle-income countries.

**Materials and Methods:**

All consecutive treatment-naïve patients with metastatic osteosarcoma were prospectively treated on the novel OGS-12 protocol consisting of sequential doublets of doxorubicin, cisplatin, and ifosfamide. Four cycles were administered as neoadjuvant therapy followed by planned curative intent surgery and metastasectomy when feasible, followed by four cycles of adjuvant chemotherapy. Baseline characteristics, histologic response, event-free survival (EFS), overall survival (OS), and toxicity data were prospectively collected.

**Results:**

Three hundred seventeen patients were enrolled onto the OGS-12 protocol from 2011 to 2014, of whom 80 (25%) had metastatic disease; median age was 17 years. The majority of patients were nutritionally challenged with high-risk features. At presentation, 83% of patients (66 patients) had lung metastases. After neoadjuvant chemotherapy, 57% of patients were histologically good responders. Four-year EFS and OS rates were 24% and 27%, respectively, in the intent-to-treat population and 27% and 29%, respectively, in the per-protocol analysis. Significant grade 3 or 4 toxicities were febrile neutropenia (51%), thrombocytopenia (36%), and anemia (54%). Histologic response was an independent predictor for EFS and OS in patients who underwent surgery. Surgical intervention was found to be significant for survival in univariable analysis.

**Conclusion:**

The novel, low-cost, non-HDMTX–based, dose-dense OGS-12 regimen has shown comparable outcomes to international standards in metastatic osteosarcomas and is worthy of wider clinical application. An aggressive multimodality approach may result in long-term survival in a select group of patients and, hence, is worth considering.

## INTRODUCTION

Prognosis is dismal in patients who have metastatic osteosarcoma at presentation, with 5-year survival estimates of only 20% to 30%.^1^ Therapy consists of aggressive surgery coupled with combination chemotherapy with or without high-dose methotrexate (HDMTX).^2,3^ A recent meta-analysis concluded that doxorubicin, ifosfamide, HDMTX, and cisplatin (but not etoposide) are active drugs in osteosarcoma, and the outcomes with three-drug regimens were far superior to those with two-drug regimens; however, the addition of a fourth drug only added to toxicity.^4^ There is no level I evidence of superiority of HDMTX-based regimens over other regimens, even in localized disease, and studies have noted higher rates of adverse events in HDMTX arms.^5,6^ The data in the metastatic setting are sparse. A Cochrane meta-analysis and international guidelines do not support HDMTX-based regimens over non-HDMTX–based combinations.^7-9^ Despite this, HDMTX-based regimens are widely used, especially in developed nations. However, administering HDMTX is challenging in resource-constrained low- and middle-income countries (LMICs) as a result of its complex interactions with many drugs and serious and unpredictable toxicity potential. Thus, complex pharmacokinetic analysis and stringent input and output monitoring are required, which add additional manpower and laboratory requirements in a costly inpatient setting.^10,11^

In the given context, a non-HDMTX–based regimen containing the three most active chemotherapeutic agents for osteosarcoma treatment was devised at our center.^12^ The provision of sequential doublets in the regimen is to make it more dose dense so as to potentiate the tumor-cell kill based on the Norton and Simon hypothesis.^13^ Dose-dense chemotherapy with antiangiogenic and proapoptotic properties has shown proof of concept in landmark trials.^14^ We report here the feasibility and outcome of this novel OGS-12 regimen in a consecutive series of patients with metastatic osteosarcoma treated at our tertiary care cancer center.

## MATERIALS AND METHODS

This was a retrospective analysis of prospectively collected data from treatment-naïve patients of all ages and sexes who had histologically confirmed, metastatic, high-grade osteosarcoma of the extremities and were treated using the novel OGS-12 regimen between November 2011 and December 2014 at our institution.

Before starting treatment, all patients had radiographs and magnetic resonance imaging of the affected area. Staging workup included noncontrast computed tomography of the thorax and bone scan. CBC, renal function (by serum creatinine and diethylenetriaminepentaacetic acid scan in some patients), liver function, echocardiography, and pure tone audiometry were performed to assess organ function. Baseline demographic features (age and sex), tumor burden markers (tumor size, lactate dehydrogenase [LDH], serum alkaline phosphatase [SAP]), and nutritional parameters (body mass index [BMI], hemoglobin, albumin, transferrin saturation, folate, and vitamin B_12_) were recorded for prognostication. Anemia was defined as hemoglobin < 12 g/dL in females and < 13 g/dL in males. Iron deficiency was defined as iron saturation < 20%, and malnourishment was defined as BMI < 18 or > 25 kg/m^2^. The nutritional parameters were monitored at baseline, after neoadjuvant chemotherapy (NACT) and adjuvant chemotherapy (ACT), and at the clinician’s discretion. Patients were also referred to a nutritionist, and supplements were given if required. Deficiencies were corrected by administering intravenous and oral formulations, as appropriate.

The chemotherapy protocol consisted of four cycles of NACT and four cycles of ACT in sequential dose-dense cycles, delivered every 21 days. NACT consisted of two cycles of doxorubicin with cisplatin followed by two cycles of doxorubicin with ifosfamide. ACT consisted of four cycles of cisplatin and ifosfamide (Data Supplement). The cumulative doses of the drugs were as follows: cisplatin 600 mg/m^2^, doxorubicin 300 mg/m^2^, and ifosfamide 54,000 mg/m^2^ over eight cycles. Primary prophylaxis with granulocyte colony-stimulating factor was used in all cycles. Planned dose reductions in subsequent cycles were based on occurrence of clinically significant hematologic and/or nonhematologic toxicity. Toxicities were documented using the National Cancer Institute Common Terminology Criteria for Adverse Events (version 4.0).^15^ After completion of NACT, patients were assessed for feasibility of surgery and underwent resection, limb salvage, or amputation as appropriate, with adequate oncologic margins. Metastasectomy was done in the same sitting or on a later date. Histologic response in the surgical specimen was assessed using necrosis grading defined by Huvo,^16^ wherein good responders were defined as those with ≥ 90% histologic necrosis. There was no change in therapy for good or poor histologic responders. Cost estimates were made by calculating the cost of chemotherapeutic drugs, investigations, admissions, and daily care charges, and comparisons were done with another international standard regimen (Data Supplement). Institutional review board permission was obtained.

### Statistical Analysis

Baseline demographic features (age and sex), tumor burden indicators (tumor size, LDH, and SAP), nutritional parameters (BMI, hemoglobin, albumin, transferrin saturation, folate, and vitamin B_12_), and toxic events (febrile neutropenia [FN], grade 3 or 4 thrombocytopenia, anemia, GI toxicity, and cardiotoxicity) were correlated with outcomes (necrosis and survival). Event-free survival (EFS) was defined as time from date of registration until progression, recurrence, or death. Overall survival (OS) was defined as time from date of registration until death from any cause or last documented follow-up. Time to progression (TTP) was defined as time from date of registration to date of progression, and postrelapse survival was defined as time from the event of progression until last documented follow-up or death. Data were analyzed using SPSS version 24.0 (SPSS, Chicago, IL). Descriptive statistics were represented as median or percentage, and group comparisons were made using the χ^2^ test or Mann-Whitney *U* test, as appropriate. Survival was estimated using the Kaplan-Meier method and compared using the log-rank test. Intent-to-treat analysis was carried out in all of the enrolled patients, and patients who were lost to follow-up (not contactable 6 months from date of analysis) were appropriately censored; in addition, per-protocol analysis was also conducted in patients who received intended NACT followed by surgery and ACT. The effect of covariates on histologic response was estimated using logistic regression analysis and on survival using Cox proportional hazards analysis. The factors found to be significant on univariable analysis were subsequently tested in multivariable analysis for all of the outcome variables.

## RESULTS

### Patient Characteristics

Between November 2011 and December 2014, 317 eligible patients were enrolled onto the OGS-12 protocol, of whom 80 patients (25%) had metastatic disease. Median age was 17 years (range, 6 to 56 years); 60 patients (75%) were male, and 20 (25%) were female (Table 1). At presentation, 53% of patients were malnourished, 44% were anemic, 48% were iron deficient, and 51% were vitamin B_12_ deficient. Mean lesion size was 11 cm, 75% of patients had high LDH, and 98% had high SAP (Table 2). Eastern Cooperative Oncology Group performance status was < 2 in 43 patients (54%) and ≥ 2 in 37 patients (46%). The most common primary tumor site was the femur (61%), and the most common histologic subtype was conventional osteosarcoma (75%). At presentation, 83% of patients (66 patients) had lung metastases, with 44% being bilateral; 8% of patients (six patients) had both lung and bone metastases, and 3% of patients (two patients) had bone metastases only at presentation. The remaining patients had metastases at other sites (eg, lymph nodes, pleural effusion). Among patients with lung metastasis, 46 patients had one to four lesions, 14 had five to eight lesions, and 12 had nine or more lesions.

**Table 1:**
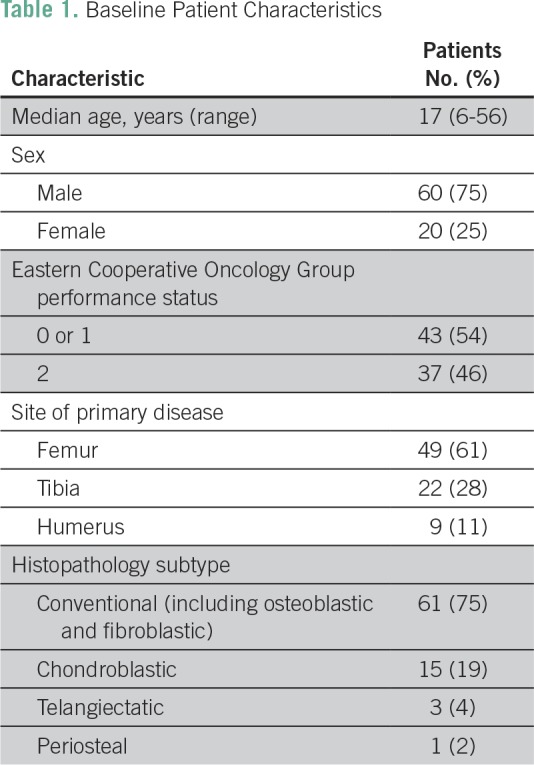
Baseline Patient Characteristics

**Table 2:**
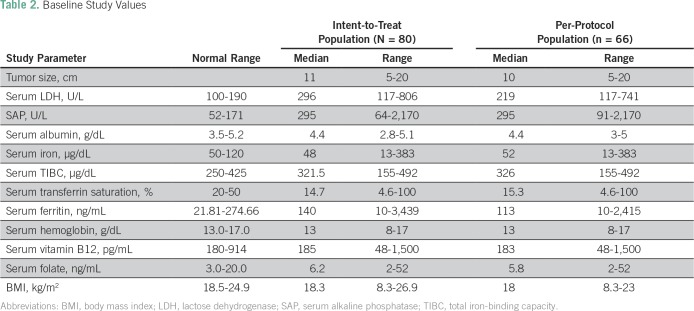
Baseline Study Values

### Histologic Necrosis

Surgery was performed in 69 patients (86%), with limb salvage in 50 patients and amputations in 19 patients, and 28 patients underwent metastasectomy at the same time or on a later date. One patient underwent extracorporeal radiotherapy. Among the 68 patients with evaluable specimens, 57% of patients had good histologic response to NACT and 22% had complete histologic response (100% necrosis). After resection, all margins were negative (Data Supplement).

### Metastasectomy

Of 69 patients who underwent surgery of the primary lesion, 28 patients (40%) also underwent metastasectomy. Among the remaining 41 patients, metastasectomies were not done as a result of (near) complete response (disappearance or < 2-mm lung nodules; n = 10), presence of pleural effusion or nodules (n = 5) or skeletal metastases (n = 2), progressive disease (increase in size of lung nodules; n = 15), or multiple bilateral lung metastases with suboptimal response (no change in size) to chemotherapy (n = 9). The final decision regarding the response to treatment was decided in a multidisciplinary clinic. Of note, patients who had stable disease after NACT underwent surgery of the primary lesion and were then continued on ACT and planned for a delayed metastasectomy. However, in some of these patients, metastasectomy could not be performed because of suboptimal response at the end of ACT.

### Intent-to-Treat Analysis of Survival

Intent-to-treat analysis was carried out in all 80 patients, and patients who were lost to follow-up were appropriately censored.

#### EFS.

At the median follow-up of 28 months (95% CI, 3 to 54 months), median EFS was 14.6 months (95% CI, 12.0 to 17.2 months). Estimated 3- and 4-year EFS rates were 24% and 24%, respectively. At median follow-up, 56 patients had disease progression. Median TTP was 10.7 months (95% CI, 1 to 25 months). Postrelapse median survival was 4.8 months (95% CI, 0 to 34 months).

#### OS.

A total of 39 deaths (48%) occurred. Median OS was 28 months (95% CI, 24.0 to 31.6 months), with 3- and 4-year OS estimates of 45% and 27%, respectively. Of 39 deaths, 37 patients died as a result of progressive disease, whereas two died of chemotherapy toxicity.

### Per-Protocol Analysis of Survival

Per-protocol analysis was carried out in 66 patients who received NACT followed by surgery and ACT of OGS-12 protocol.

#### EFS.

**Fig 1:**
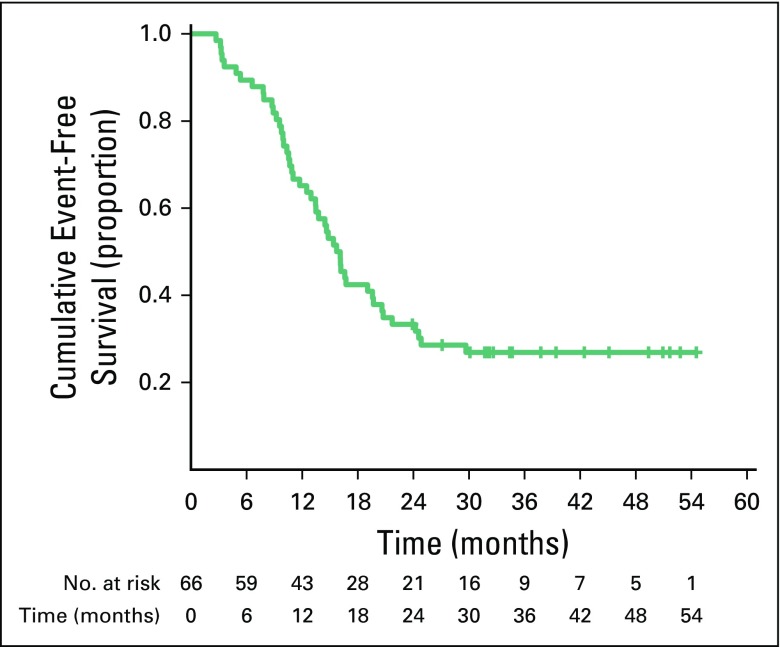
Event-free survival in per-protocol population (n = 66).

At median follow-up of 32 months (95% CI, 8 to 55 months), median EFS was 16 months (95% CI, 13.5 to 18.0 months). Estimated 3- and 4-year EFS rates were 27% and 27%, respectively (Fig 1). At median follow-up, 47 patients had disease progression. Median TTP was 13 months (95% CI, 3.0 to 25.0 months), and median postrelapse survival was 5 months (95% CI, 0 to 34 months).

#### OS.

**Fig 2:**
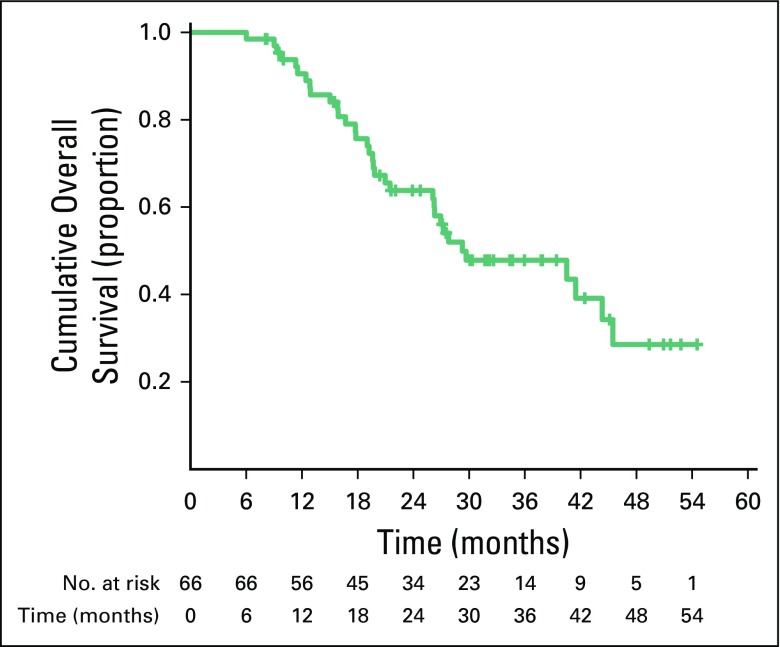
Overall survival in per-protocol population (n = 66).

Median OS was 29 months (95% CI, 15.0 to 43.4 months). Three- and 4-year estimates of OS were 48% and 29%, respectively (Fig 2). At median follow-up, a total of 34 deaths occurred as a result of progressive disease.

### Toxicity

Of 80 patients, 41 (51%) developed FN. Of a total of 512 cycles administered, 64 episodes (13%) of FN occurred. Other grade 3 or 4 hematologic toxicities were anemia and thrombocytopenia, which were seen in 43 patients (54%) and 29 patients (36%), respectively. Three patients (4%) developed grade 3 or 4 serum creatinine elevation, and three patients (4%) developed grade 2 heart failure. A decrease in glomerular filtration rate > 20% by diethylenetriaminepentaacetic acid was documented in five patients (6%), and an asymptomatic decrease in left ventricular ejection fraction of ≥ 10% on two-dimensional echocardiography was reported in 15 patients (19%). This decrease subsequently recovered in 14% of patients. Grade 1 or 2 hearing loss was seen in two patients (3%). Dose reduction was required in 16 patients (20%).

### Factors Correlating With Outcomes

On univariable and multivariable analysis, histologic response was found to be significantly correlated with both EFS and OS in both of the analyses. Whether surgery was performed was found to be significantly correlated with EFS and OS in univariable analyses. However, it lost significance in multivariable analysis.

## DISCUSSION

The goal of bone sarcoma therapy is curing the patient of both the primary tumor and all metastatic deposits while maintaining maximal extremity function and minimizing treatment-specific, late adverse effects. Because of the lack of any definitive evidence of superiority of one regimen over the other in osteosarcoma, oncologists use HDMTX-based or non-HDMTX–based regimens depending on institutional practices and availability.^5,6^ Chemotherapy response, metastasis, and tumor size and site are reported to be independent prognostic factors of osteosarcoma.^17^ In addition, it has been suggested that vascular endothelial growth factor expression, dynamic contrast-enhanced magnetic resonance imaging, and positron emission tomography–computed tomography parameters are associated with prognosis.^18-20^

Our OGS-12 protocol, which is a three-drug non-HDMTX regimen, resulted in good histologic response in 57% of patients, which is comparable to the good response rates seen in the studies by Goorin et al^21^ (65%) and Bacci et al^22^ (54%). The per-protocol analysis regarding survival has been included because it is important to know (especially in LMICs, where compliance is multifactorial) the outcome of the patients who have completed the intended treatment. The ability to complete the intended treatment has been found to have bearing on the outcomes.^23^ Goorin et al^21^ and Bacci et al^22^ both reported OS rates of 55%, whereas a study from the Memorial Sloan Kettering Cancer Center^24^ reported 11% OS at 2 years. Bielack et al,^17^ Kager et al,^25^ and Mialou et al^26^ reported 5-year OS rates of 31.6%, 29%, and 19%, respectively, in patients with metastatic osteosarcoma. Daw et al,^27^ from St Jude, found 5-year survival rates of 45.5% and 8.3% in patients with lung metastasis only treated with the OS-86 protocol (12 patients receiving ifosfamide, cisplatin, doxorubicin, and HDMTX) and OS-91 protocol (17 patients receiving ifosfamide, carboplatin, doxorubicin, and HDMTX), respectively. Our study found 3- and 4-year OS rates of 45% and 27%, respectively, using the a novel OGS-12 protocol in a patient population with high tumor burden and nutritional deficiencies; these results are comparable to international standards. The EFS rates were also comparable to those found in international studies (Table 3). The outcomes of this protocol in nonmetastatic patients are also comparable to international standards.^12^

**Table 3:**
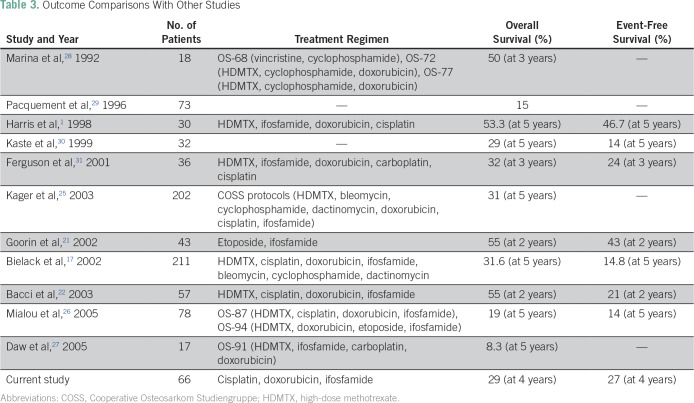
Outcome Comparisons With Other Studies

Toxicities as a result of treatment have not been mentioned in the studies by the Memorial Sloan Kettering Cancer Center,^24^ Bielack et al,^17^ Daw et al,^27^ Kager et al,^25^ and Mialou et al.^26^ Bacci et al^22^ reported a renal toxicity rate of 21%, probably attributed to the use of HDMTX in their protocols. Toxicities in our study have been compared with those in the study by Goorin et al^21^ in Table 4. In the study by Goorin et al,^21^ FN incidence was not reported; in our study, there was a higher incidence of anemia, which is partially explained by the fact that our patients had a high incidence of anemia and nutritional deficiencies at baseline.

**Table 4:**
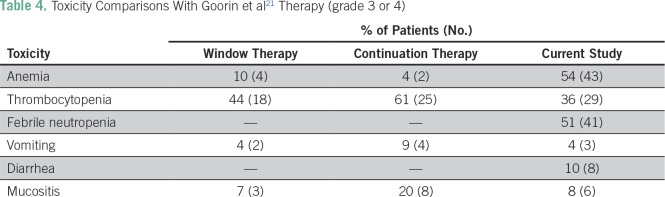
Toxicity Comparisons With Goorin et al^21^ Therapy (grade 3 or 4)

Our analysis indicated that histologic response to NACT was an independent predictor of both EFS and OS in both the per-protocol and intent-to-treat analyses, which is also concordant with other studies.^17,32-34^

Of note, in univariable analysis, the patients in whom surgery could be done fared better than patients who could not undergo surgery. However, this lost significance in multivariable analysis, perhaps because of the small numbers and relatively short follow-up time. Selection bias might also be a possibility. Furthermore, there is a complex interplay of various factors including disease biology that might dilute the impact of surgery. Patients were subjected to metastasectomy with an attempt to remove the lesions maximally, where feasible. Interestingly, the 25% of patients (16 of 66 patients) who had no event up to the 30-month period continued to remain event free afterward. This highlights the importance of an aggressive approach in patients with metastatic osteosarcoma consisting of combination chemotherapy with surgery of the primary tumor and resection of all the metastatic sites. A subset of patients can derive long-term survival with this approach, and this is meaningful considering the otherwise dismal survival in this population. Results of few other studies indicate that resectable metastases should be maximally removed, regardless of their location and number. There is a five-fold increase in the risk of dying in patients with a clinically detected tumor burden when compared with those who have had a complete resection.^25,35^ The observations noted in other malignancies, such as advanced renal cell cancers and ovarian cancers, have shown benefit with primary surgery,^36,37^ whereas in breast cancer, one trial showed benefit while the other trial did not support surgical intervention of primary tumor in metastatic disease.^38,39^

HDMTX administration warrants drug monitoring, which may not be routinely available across all parts of the world. Furthermore, HDMTX is known for its unpredictable toxicity pattern and complex interactions with other chemotherapeutic and supportive drugs.^10,40^ Hypoalbuminemia can increase the clearance time of HDMTX, leading to hyperbilirubinemia and prolonged hospitalization.^41^ Lack of facilities for methotrexate level monitoring, high infection rates, underlying malnutrition, and inadequate supportive care facilities make it difficult to administer HDMTX to most of the patients with osteosarcoma in India and other LMICs. In addition, the costs of a protocol incorporating HDMTX are much higher compared with our protocol (Data Supplement). Hence, the OGS-12 protocol offers a practical, economical, and effective alternative in countries with poor resources such as India.

The strengths of this study include the use of a novel, dose-dense, non-HDMTX–based combination chemotherapy protocol, OGS-12, in a uniform manner. This was tested prospectively in a successive cohort of 80 treatment-naive patients with metastatic osteosarcoma over a comparatively short period of 3 years in a single center. Thus, the results of this study are widely applicable to most patients presenting to resource-constrained centers in LMICs. Inpatient treatment with cumbersome pharmacokinetic monitoring may not be available to all such centers. The toxicities associated with our protocol also have been listed in detail, whereas many other comparable studies have not reported toxicities. In addition, to the best of our knowledge, our study is the only one from Asia reporting outcomes of patients with metastatic osteosarcoma and one of only a few studies worldwide available for this rare disease. Notably, we have chosen treatment-naïve patients because prior inadvertent treatment is a significant confounding factor and affects prognosis.^42^

Further, our analysis also suggests that patients with osteosarcoma in India present with high tumor burden, which could be attributed to delayed presentation to the health care system. This is true for other LMICs as well. Further studies are needed to address the social and logistical issues and to research the pharmacogenomic and biologic differences in these tumors.

The weaknesses of this study were the relatively short follow-up and its single-arm, nonrandomized nature. Of note, it is difficult to conduct randomized studies in osteosarcoma, especially in the metastatic population, because it is rare. Furthermore, such rare tumors have shown tremendous progress based on many sequential, single-arm studies.

In conclusion, treatment with the novel OGS-12 protocol, a dose-dense, non-HDMTX–based sequential doublet regimen, produces outcomes in treatment-naïve patients with metastatic osteosarcoma comparable to international standards with acceptable toxicity and merits wider clinical application. The aggressive approach of intensive combination chemotherapy, surgery, and metastasectomy may result in long-term survival in a select group of patients and is thus worth considering.
